# SARS-COV-2 spike protein promotes RPE cell senescence via the ROS/P53/P21 pathway

**DOI:** 10.1007/s10522-023-10019-0

**Published:** 2023-02-04

**Authors:** Yuhang Zhang, Xuyan Peng, Mengjiao Xue, Jingjing Liu, Guohui Shang, Mingjun Jiang, Dandan Chen, Baixue Liu, Yuxuan Wang, Xiaolin Jia, Jianqing Xu, Fengyan Zhang, Yanzhong Hu

**Affiliations:** 1 The Laboratory of Ophthalmology and Vision Science, Department of Ophthalmology, The First Affiliated Hospital of Zhengzhou University, Zhengzhou, China; 2 Joint National Laboratory for Antibody Drug Engineering, The First Affiliated Hospital of Henan University, Kaifeng, China; 3 Kaifeng Key Lab for Cataract and Myopia, Institute of Eye Disease, Kaifeng Central Hospital, Kaifeng, China; 4 Department of Medical Genetics and Cell Biology, School of Basic Medical Sciences, Zhengzhou University, Zhengzhou, China; 5 Chongqing Institutes for Life Science Innovation; Clinical Center for Bio-Therapy, Zhongshan Hospital & Institutes of Biomedical Sciences, Fudan University, Shanghai, 200032 People’s Republic of China; 6 Department of Cell Biology and Genetics, School of Medicine, Henan University, Jin-Ming Road, Kaifeng, 475014 China; 7 The Division of Ophthalmology and Vision Science, Department of Ophthalmology, The First Affiliated Hospital of Zhengzhou University, Zhengzhou University, No.1 Long-Hu-Zhong Huan Road, Zhengzhou, China

**Keywords:** Spike protein, SARS-Cov-2, RPE, Zebrafish, Senescence

## Abstract

SARS-Cov-2 infection, which has caused the COVID-19 global pandemic, triggers cellular senescence. In this study, we investigate the role of the SARS-COV-2 spike protein (S-protein) in regulating the senescence of RPE cells. The results showed that administration or overexpression of S-protein in ARPE-19 decreased cell proliferation with cell cycle arrest at the G1 phase. S-protein increased SA-β-Gal positive ARPE-19 cells with high expression of P53 and P21, senescence-associated inflammatory factors (e.g., IL-1β, IL-6, IL-8, ICAM, and VEGF), and ROS. Elimination of ROS by N-acetyl cysteine (NAC) or knocking down p21 by siRNA diminished S-protein-induced ARPE cell senescence. Both administrated and overexpressed S-protein colocalize with the ER and upregulate ER-stress-associated BIP, CHOP, ATF3, and ATF6 expression. S-protein induced P65 protein nuclear translocation. Inhibition of NF-κB by bay-11-7082 reduced S-protein-mediated expression of senescence-associated factors. Moreover, the intravitreal injection of S-protein upregulates senescence-associated inflammatory factors in the zebrafish retina. In conclusions, the S-protein of SARS-Cov-2 induces cellular senescence of ARPE-19 cells in vitro and the expression of senescence-associated cytokines in zebrafish retina in vivo likely by activating ER stress, ROS, and NF-κb. These results may uncover a potential association between SARS-cov-2 infection and development of AMD.

**Supplementary Information:**

The online version contains supplementary material available at 10.1007/s10522-023-10019-0.

## Introduction

Age-related macular degeneration (AMD) affects approximately 0.4–8.7% of people over the age of 50 (Jonas et al. 2017) and is a leading cause of blindness among the elderly in the developed world (Bourne et al. 2013). The majority of affected individuals (about 80–85%) suffer from dry AMD, characterized by atrophy of photoreceptor cells, ganglia cells, and retinal pigment epithelial cells (RPE). An increase in the number of senescent cells in the RPE is associated with early onset AMD. This can be triggered by multiple risk factors such as UV and blue light radiation, hyperglycemia, retina ischemia, and viral infections (Urbanelli et al. 2016; Upadhyay et al. 2020; Cheng et al. 2021). The severe acute respiratory syndrome coronavirus 2 (SARS-CoV-2) infection, which attacks multiple tissues including the eye fundus(Marinho et al. 2020), can trigger cellular senescence in infected cells (Maremanda et al. 2020, Meyer et al. 2021; Tripathi et al. 2021). This implies that SARS-CoV-2 may be associated with retinopathy.

SARS-CoV-2 is a positive sense, single-stranded, enveloped, and non-segmented RNA virus that belongs to the coronaviridae family (Guan et al. 2020), and its infection has resulted in a pandemic around the world (Guan et al. 2020, Hoffmann et al. 2020; Lani-Louzada et al. 2020; Zhou et al. 2020). SARS-CoV-2 infects the target tissues via the S-protein and its interaction with the angiotensin-converting metallopeptidase 2(ACE2) on receptor cells (Hoffmann et al. 2020). S-protein is proteolytically cleaved by cellular cathepsin L and the transmembrane protease serine 2(TMPRSS2) (Heurich et al. 2014; Song et al. 2018). ACE2 is widely expressed in most tissues including the respiratory system, eye, and gastrointestinal mucosa (Dimitrov 2003; Li et al. 2003; Bonn 2004; Hamming et al. 2004). Infection of SARS-CoV-2 causes an acute inflammation, which can result in organ dysfunction, such as lung collapse (Guan et al. 2020). It also can promote senescence in infected cells and increase their expression of ACE2, which can in turn increase SARS-CoV-2 infection (Camell et al. 2021; Duarte et al. 2021; Meyer et al. 2021; Tripathi et al. 2021). During SARS-CoV-2 infection, S-protein is cleaved into S1 and S2 subunits. The receptor-binding domain in the S1 subunit binds to ACE2. The heptad repeat 1(HR1) and 2(HR2) domains in the S2 subunits interact with each other to form a six-helix bundle (6-HB) fusion core, bringing viral and cellular membrane into proximity for fusion and infection(Yan et al. 2020). The S-protein of SARS-CoV-2 is associated with cellular senescence of the lung (Maremanda et al. 2020), endothelial cells (Duarte et al. 2021; Meyer et al. 2021)and macrophages (Duarte et al. 2021). SARS-CoV-2 virion and its S-protein promote cellular senescence by partially upregulating interferon-γ and toll-like receptor (Meyer et al. 2021; Tripathi et al. 2021).

The eye is a potential window for transmission of SARS-CoV-2 (Reinhold et al. 2021; Wan et al. 2021). ACE2 is expressed in the cornea, conjunctiva, iris, RPE, and retinal capillary (Marinho et al. 2020; Araujo-Silva et al. 2021; Martin et al. 2021). SARS-CoV-2 viral particles are found in the retina of postmortem donors (Marinho et al. 2020) and associated with retinopathies such as hyperreflective lesions at the level of the ganglion cells and inner plexiform layers, subtle cotton wool spots, and visible microhemorrhages (Virgo and Mohamed 2020; Araujo-Silva et al. 2021). However, the association of retinopathy and SArS-CoV-2 infection remain unclear.

In this paper, we studied the effect of S-protein of SARS-CoV-2 on ARPE-19 cells in vitro and on the zebrafish retina in vivo. We found that ARPE-19 cells express ACE2. Overexpression of S-protein cDNA or administration of purified S-protein attenuates ARPE-19 cell proliferation with cell cycle arrest in the G1 phase. S-protein can activate ROS, ER stress and NF-κB in ARPE-119 cells, and promote cellular senescence. The transient intravitreal injection of S-protein upregulates the senescence-associated inflammatory factors in the zebrafish retina. These results suggest SARS-CoV-2 infection may lead to chronic retinopathy, including AMD.

## Methods

### Chemical reagents and antibodies

The SARS-CoV-2 S-protein was bought from Novoprotein Scientific (Shanghai, China). NAC(N-Acetylcysteine) and BAY-11-7082 were from Med-Chem Express (New Jersey, USA). The rabbit antibody to ACE2 was from Abcam (Cambridge, MA, USA). The antibodies against P21, P53, BCL-2, IκB, CHOP, ATF4, ATF6, Brg-1, and SARS-CoV-2/S-protein were from Cell Signaling Technology (Shanghai, China). The antibodies against BIP, Calnexin, GAPDH, β-actin, and P65 were from Proteintech (Wuhan, China). The antibody against ATF3 and siRNA of p21cip1 were from Sigma-Aldrich (St. Louis, MO, USA). The senescence-associated β-galactosidase staining kit was bought from Beyotime Institute of Biotech (Shanghai, China). The transfection reagent Lipofectamine3000 was from Invitrogen (Carlsbad, CA, USA).

### Plasmids

Plasmids p3xflag-cmv-7.1, pQCXIP-GFP1-10, pQCXIP-BSR-GFP11, pCMV-ACE2-Myc, and pDsRed2-ER were ordered from Addgene (MA, USA). Plasmid pSV-wuhan Spike was gifted by Dr. Jianqing Xu from Fudan Institute of Biomedical Research, Shanghai.

p3xflag-S was generated using the cDNA of S-protein amplified from the pSV-wuhan Spike plasmid with primers (Table 1). The PCR products were subcloned into the p3xflag-cmv-7.1 vector at the SalI and HindIII restriction sites, generating p3xFlag-S. All recombinant plasmids were verified by DNA sequencing.

**Table 1:** The primers for detecting P21, P53, and senescence-associated inflammation factors

Name	Forward (5′ to 3′)	Revers (5′ to 3′)
IL-1β	AGTACCTGAGCTCGCCAGT	TGGTGGTCGGAGATTCGTAG
IL-6	TGAACTCCTTCTCCACAAGCG	CCGTCGAGGATGTACCGAAT
IL-8	GCTCTGTGTGAAGGTGCAGTT	ACCCAGTTTTCCTTGGGGTC
MCP-1	CGCCTCCAGCATGAAAGTCT	AGGTGACTGGGGCATTGATT
P21	AAGTCAGTTCCTTGTGGAGC	GCCATTAGCGCATCACAGTC
P53	ACCTATGGAAACTACTTCCTGAA	CTGGCATTCTGGGAGCTTCA
Bcl-xl	CCTAAGGCGGATTTGAATAATCTT	CCAAAACACCTGCTCACTCAC
VEGFA	CCCACTGAGGAGTCCAACATC	CTGCATTCACATTTGTTGTGCTG
ICAM	GGTAGCAGCCGCAGTCATAA	GATAGGTTCAGGGAGGCGTG
MMP3	AGCCAACTGTGATCCTGCTT	CCACGCCTGAAGGAAGAGAT
TGF-β	GGAAATTGAGGGCTTTCGCC	CCGGTAGTGAACCCGTTGAT
P14	CGCGAGTGAGGGTTTTCGTG	AGTAGCATCAGCACGAGGG
GAPDH	GACAGTCAGCCGCATCTTCT	GCGCCCAATACGACCAAATC
Spike	CCCAAGCTTTTCGTGTTCCTGGTGCTC	CCCGTCGACTCAAGCGTAATCTGGAACATCGTATGGGTAGGTGTAGTGGAGCTTCACGCC
z-p53*	GTACTTGCCGGGATCGTTTG	GCGGGAACCTGAGCCTAAAT
z-p21*	TTCAGGTGTTCCTCAGCTCCT	GTGAACGTAGGATCCGCTTGT
z-p16*	TGATCTACACAGCCACAGGA	GCAGTGAGTTTGTTGCCTCTC
z-il1b*	CTGAAATGATGGCATGCGGG	TGCAAGCGGATCTGAACAGT
z-il6*	CCTCAGTCCTGGTGAACGAC	GAACAGGATCGAGTGGACCG
z-il8*	GAAAGCCGACGCATTGGAAA	TTAACCCATGGAGCAGAGGG
z-icam1*	AGGAAACTACACCTGCACCG	CGTGGATGGAACCACCAGAA
z-vegfa*	TGCTGTAATGATGAGGCGCT	CATCTTGGCTTTTCACATCTTTCT
z-bip*	ACCACATACTCCTGTGTTGGAG	ATGACGGAGTGATGCGGTTT
z-chop*	AGGACACGTAGAGAAGGGGAC	TCCGTTGAGCTCCACATTCTT
z-atf3*	TTGGGTCCGTCAGAGATCAGT	GGTCGTTCTCCTCTGGGACA
z-atf6*	TACGCTCCTCACCGAACCTA	GGACCACTGACATCTTGGGG
z-atf6b*	AGACGCATCGCTCCATTGAA	GGTCGATGGCGTCTAGGAAG
z-ef1a*	AAGATCGGCTACAACCCTGC	TTCCATCCCTTGAACCAGCC

*z zebrafish

### Zebrafish

Danio rerio zebrafish were maintained at 26–28.5 °C in a circulating water system with a light–dark cycle of 14:10 h (h) according to the zebrafish husbandry protocol. The fish were fed three times daily with newly hatched brine shrimp. The developmental stages were determined by month post fertilization (mpf). Handling of all animals used in this project followed the guidelines for use of animals in ophthalmic and vision research. The procedures used in our study were approved by the ethics committee of The First Affiliated Hospital, Zhengzhou University.

### Cell culture and cell transfection

ARPE-19 cells were cultured in DMEM/F12 media containing 10% FBS with 1 × ampicillin and streptomycin. HEK293 cells were maintained in our lab and cultured in DMEM media containing 10% FBS with 1× ampicillin and streptomycin.

For transfection, ARPE-19 or HEK293 cells were seeded on 6-well cell culture plates overnight. The cells were transfected with plasmids with Lipofectamine3000 following the protocol with kit.

### The cell syncytia

293 T cells were seeded at approximately 50% confluence in a six-well plate overnight. The cells were transfected individually with p3xflag-S or pCMV-ACE2-Myc. After transfection, the cells were trypsinized. HEK293/p3xflag-S cells were mixed with HEK293/pCMV-ACE2-Myc cells at a ratio of 1:1, and the mixed cells were cultured for 24–48 h. The multinucleated giant cells were photographed.

The bimolecular fluorescence complementation (BiFC) assay (Kodaka et al. 2015): HEK 293 cells were transfected either with p3xflag-S + pQCXIP-GFP-11 or pCMV-ACE2-Myc plus pQCXIP- GFP1-10. After transfection, the cells were trypsinized, mixed, and cultured for another 24 h. GFP positive cells were photographed and the GFP signal was used as a marker of the cell syncytia.

### Cell proliferation

The proliferation of ARPE-19 cells that were treated with 0.5 ng/ml S-protein for up to 96 h was measured in a real-time cell analyzer (RTCA, Roche-Applied Science, GmbH, Penzberg, Germany). 2 × 10^3^ cells were seeded in an E-plate 16 in 200 μl of F12/DMEM complete media, and cultured in 5% CO2 incubator at 37 °C for up to 120 h. The cell index (CI) was obtained to assess for cell proliferation using the RTCA software.

### Flow cytometry

For cell cycle analysis, 1 × 10^6^ cells were collected and fixed with 70% ethanol. The cells were stained with PI solution with RNase A (BeyoTime, Shanghai, China) for 30 min and analyzed on the FACS cytometry machine (BD Biosciences). The data were analyzed using the FlowJo software (Tree Star).

For ROS analysis, cellular ROS was quantified using the cellular ROS Detection Assay Kit from Solarbio (Beijing, China). The cells were seeded in 60 mm plates at 1 × 10^6^ cells. After treatment, the cells were incubated with DCFH-DA for 30 min at 37 °C. ROS signal was measured and analyzed using flow cytometry.

### Intravitreal injection of S-protein zebrafish

S-protein (6.3 ng/100 nl PBS) was injected into the vitreous humor of the 7 mpf zebrafish. After recovery for 2 and 3 days post injection, the zebrafish were euthanized. The retina was fixed in 4% Paraformaldehyde overnight followed by cryosection. The tissue slide was 8 μM thickness. H&E staining was performed following the protocol provided with the kit.

### Immunoblotting and immunofluorescent staining

For immunoblotting, 30–50 µg of lysates were separated by sodium dodecyl sulfate–polyacrylamide gel electrophoresis, and the proteins were transferred to polyvinylidene difluoride membranes. After blocking, the membranes were incubated with primary antibodies at 4 °C overnight. The membranes were washed and incubated with secondary antibodies. The The signals were detected by a CCD camera-based imager (Amersham Imager 680, GE).

For immunofluorescent staining, the treated ARPE-19 cells were fixed in 3.7% Polyformaldehyde/PBS for 20 min, followed by permeabilization in 0.5% triton x-100 for 2 min. The cells were then incubated with 2% BSA/PBS block buffer for 1 h followed by incubation with primary antibodies for 1 h. The cells were washed and then incubated with secondary antibody 1 h. After mounting in buffer containing DAPI for nuclear staining, the fluorescent signals were photographed with a confocal microscope (Arial, Japan).

### SA-ß-Gal staining assay

ARPE-19 cells were seeded into 12-well plates and incubated overnight. Cells were treated with 0.5 ng/ml S-protein for 48 h or transfected with a p3xflag-S recombinant plasmid, followed by recovery in complete medium for 48 h. The cells were fixed in a buffer containing 0.5% glutaraldehyde and 2% paraformaldehyde in PBS for 20 min. After this, the cells were incubated with a solution containing X-gal for 12 h at 37 °C to measure SA-ß-Gal activity. SA-ß-Gal positive signals were photographed under the microscope (Zeiss, Oberkochen, Germany). The number of SA-ß-Gal positive cells was normalized to the total number of cells. The data represent mean ± SD, N = 3. The Student’s T-test was used for statistical analysis. *p < 0.05; **p < 0.01.

### Quantitative real-time PCR (qRT-PCR)

Total RNA was extracted with RNAiso reagent following the manufacturer's protocol (Takara, Beijing, China). One microgram of total RNA was used to synthesize cDNA (Takara). Equal amounts of cDNA were mixed with Fast start Universal SYBR Green Master Mix (Roche, San Francisco, CA, USA). qRT-PCR was performed using an ABI 7500 system (Applied Biosystems, Foster City, CA, USA). The primers for detecting P21, P53, and senescence-associated inflammation factors are listed in Table 1.

### Statistical analysis

All data are presented as mean ± SD and analyzed using the SPSS v17.0 (SPSS Inc.). The experiments were performed in triplicate. One-way ANOVA followed by Tukey's post hoc test was used to compare the differences between continuous data (> 3 groups), while differences between 2 groups were compared using an unpaired Student's t-test. *p < 0.05 denotes significance.

## Results

### SARS-CoV-2 S-protein induces the senescence of RPE cells in vitro

Infection of SARS-CoV-2 or pseudo-typed virus expressing spike protein induces senescence in human kidney endothelial and lung epithelial cells (Schuler et al. 2021, Tripathi et al. 2021). Since the SARS-CoV-2 virion was identified in postmortem retina tissues and retinal RPE cells express ACE (Martin et al. 2021; Reinhold et al. 2021), we proposed that S-protein was associated with RPE cellular senescence. To do this, we firstly validated the activity of S-protein. The HEK293 containing plasmidp3xflag-S were cocultured with PCMV-HA-ACE2 -containing HEK293 cells (Fig. S1, A) and the giant fusion cells were observed (Fig. S1, C). This S-protein-mediated cell fusion was also confirmed by using the bimolecular fluorescence complementation (BiFC) assay (Kodaka et al. 2015) (Fig. S1, D, materials and methods). Co-culturing the HEK293 cells with pGFP1-10 plus p3xflag-S with HEk293 cells containing pGFP-11 + CMV-ACE2-HA produced GFP positive cells (Fig. S1, E). No GFP signal was observed in the cells that expressed Flag-S + GFP1-10 or ACE2 + GFP-11 alone (Fig. S1, E). These results suggested that the expressed Flag-S-protein is active and able to induce ACE2- expressed HEK293 cell fusion.

**Fig. 1:**
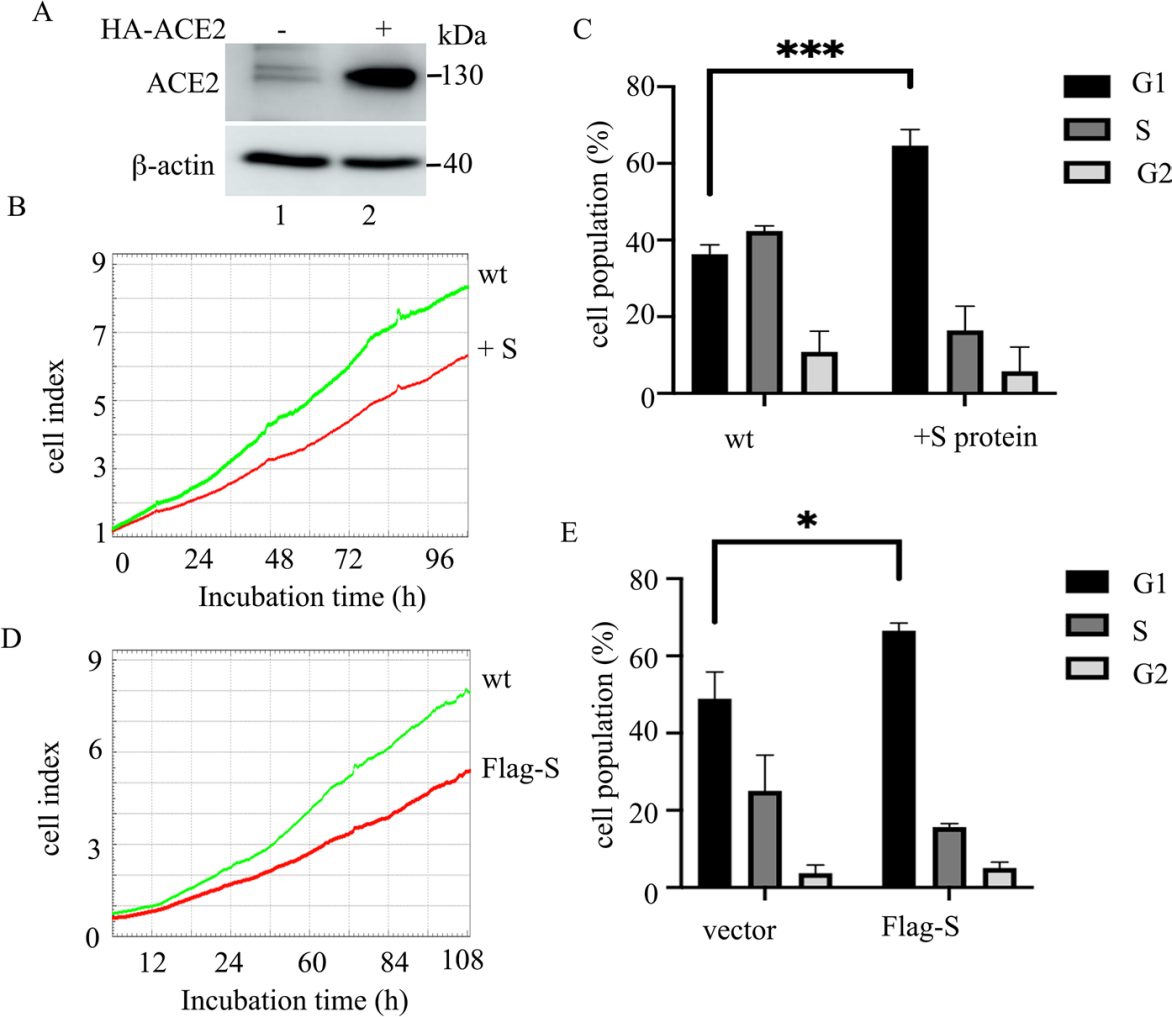
SARS-CoV-2 S-protein inhibits ARPE-19 cell proliferation in vitro. **A** Immunoblot of ACE2 and β-actin in ARPE-19 or HA-ACE2-overexpressed ARPE-19 cells. **B** Real-time cell analyzer (RTCA) detection of cell proliferation of ARPE-19 cells (wt), S-protein-treated ARPE-19 cells (**B**), or Flag-S overexpressed ARPE-19 cells (**D**). **C** and **E** Flow cytometry measurement of the cell cycle of ARPE-19 cells (wt), S-protein-treated ARPE-19 cells (+ S-protein), or Flag-S-overexpressed ARPE-19 cells (Flag-S). The black, gray and blank rectangles represent G1, S, and G2 phases respectively. The data represent mean ± SD, n = 3. Unpaired Student’s T-test was used for statistical analysis. *p < 0.05

**Fig. 2:**
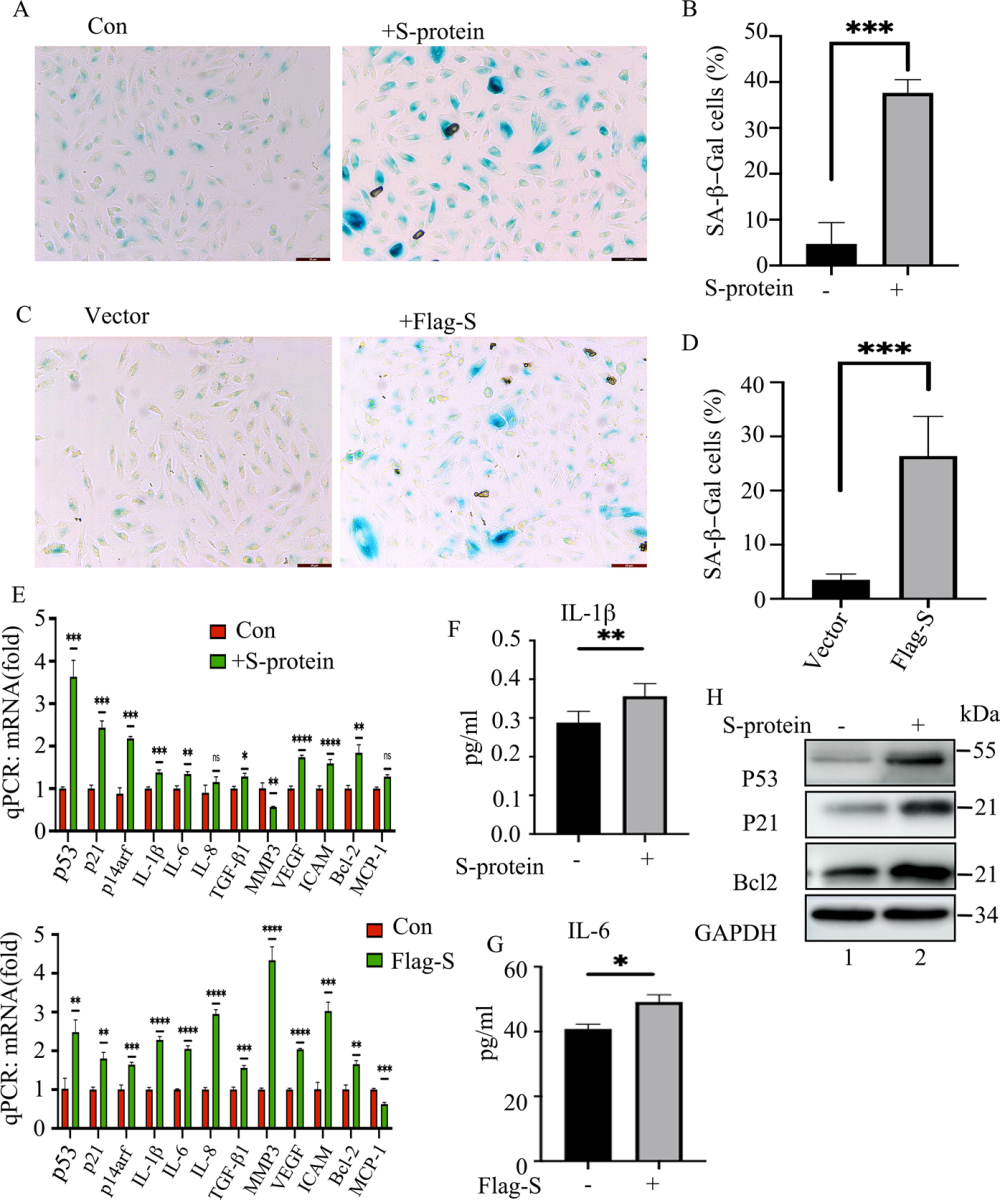
S-protein induces senescence of ARPE-19 cells. **A** SA-β-Gal staining of ARPE-19 cells that were treated with media containing PBS (Con) or S-protein for 48 h. **B** Quantitation of the percentage of SA-β-Gal positive cells per view in **A**. Ten views were used for quantitation, and the experiment was repeated three times. The data represent mean ± SD, n = 3, ***p < 0.001. **C** SA-β-Gal staining of the ARPE-19 cells that express empty vector or p3xFlag-S respectively for 48 h. **D** Quantitation of the percentage of SA-β-Gal positive cells/ total cells/view (a total of 10 views were used for quantitation). The data represent mean ± SD (n = 3), ***p < 0.001. **E** qPCR to quantitate the expression of cell cycle inhibitors (P53, p21, and p14arf), cytokines (IL-1β, IL-6, IL-8, MCP-1, TGF-β1), MMP3, and iCAM in the cells treated in **A** and **C**. **F** and **G** ELISA to measure the secretion of IL-1β and IL-6 in ARPE-19 cells that were treated with S-protein for 48 h. **H** Immunoblot of the expression of p53, p21, Bcl-2, and GAPDH in ARPE-19 cells that were treated with S-protein for 48 h

**Fig. 3:**
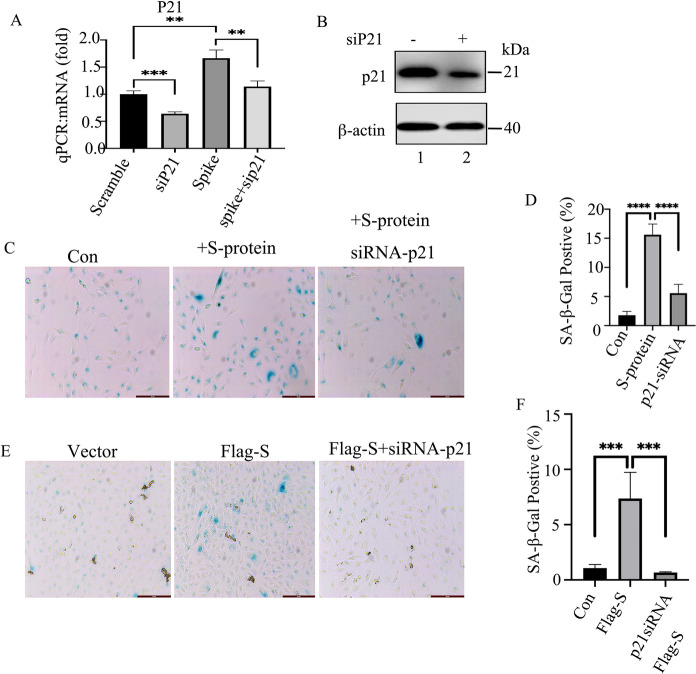
Knocking down p21 by siRNA reduces S-protein-induced cell senescence. **A** qPCR to measure the expression of p21 in ARPE-19 cells expressing scramble siRNA or siRNA to P21 followed by treatment with S-protein for 48 h. The data represent mean ± SD, (n = 3), **p < 0.01, ***p < 0.001. **B** Immunoblot of p21 in ARPE-19 cells that expressed scrambled siRNA or siRNA against p21. **C** SA-β-Gal stain in ARPE-19 cells that were transfected with scramble or siRNA-P21 followed by treatment with S-protein for 72 h. **D** Quantitation of the percentage of SA-β-Gal positive cells vs total cell number per view in **C**. The data represent mean ± SD, (n = 3), **p < 0.01, ***p < 0.001. **E** SA-β-Gal stain assay, ARPE-19 cells were transfected with empty vector, Flag-S and Flag-S + siRNA-P21 for 72 h. **F** Quantitation of the percentage of SA-β-Gal positive cells vs total cell numbers per view in **E**. The data represent mean ± SD, (n = 3), ***p < 0.001

To determine the role of S-protein in RPE cells, we transfected p3xflag-S into ARPE-19 cells. After 48 h recovery, the regulatory effects of Flag-S-protein on ARPE-19 cells were studied. The results showed that ARPE-19 cells expressed low levels of ACE2 at baseline (Fig. 1A, lane 1 and 2). Ectopic expression of Flag-S-protein attenuated ARPE-19 cells’ proliferation (Fig. 1D) and blocked cell cycle at the G1phase (Fig. 1C). In addition, we treated ARPE-19 cells with purified S-protein for up to 96 h and the cell proliferation was tested. As the results indicated that the administrated S-protein could inhibit ARPE-19 cells’ proliferation (Figs. 1B, E, and S2A). Both ectopic expression and administration of S-protein caused a low degree of cellular apoptosis (data not shown). To test whether S-protein induces cellular senescence, we mearsured that the senescence-associated biomarkers in the 48 h-Flag-S-overexpressed cells or 48 h-S-protein-treated cells (Fig. 2) (Faragher 2021). Ectopic Flag-S or direct administration of S-protein induced cell senescence, such as increasing the number of SA-β-Gal positive cells (Fig. 2A–D), the expression of 53, p21, and p14ARF at the protein and mRNA levels (Fig. 2E and H), the expression of senescence-associated inflammatory factors (like IL-1β, IL-16, IL-8, TGF-β1, VEGFA, and iCAM) (Fig. 2E) and cell survival factor BCL-2 (Fig. 2E and H). The ELISA results indicated that administration of S-protein increased the secretion of IL-1β and IL-6 in ARPE-19 cells (Fig. 2F and G). But the expression of another two senescent cytokines of MMP-3 and MCP-1 varied between the Flag-S-expressed cells and S-protein-treated cells with unknown mechanism (Fig. 2E). Knocking down p21 by siRNA (Fig. 3A and B) reduced the number of S-protein-induced SA-β-Gal positive ARPE-19 cells (Fig. 3C–F). Taken together, these results suggested that S-protein induced ARPE-19 cell senescence by partially activating the p53-p21 pathway.

### S-protein increases ROS production in ARPE-19 cells

**Fig. 4:**
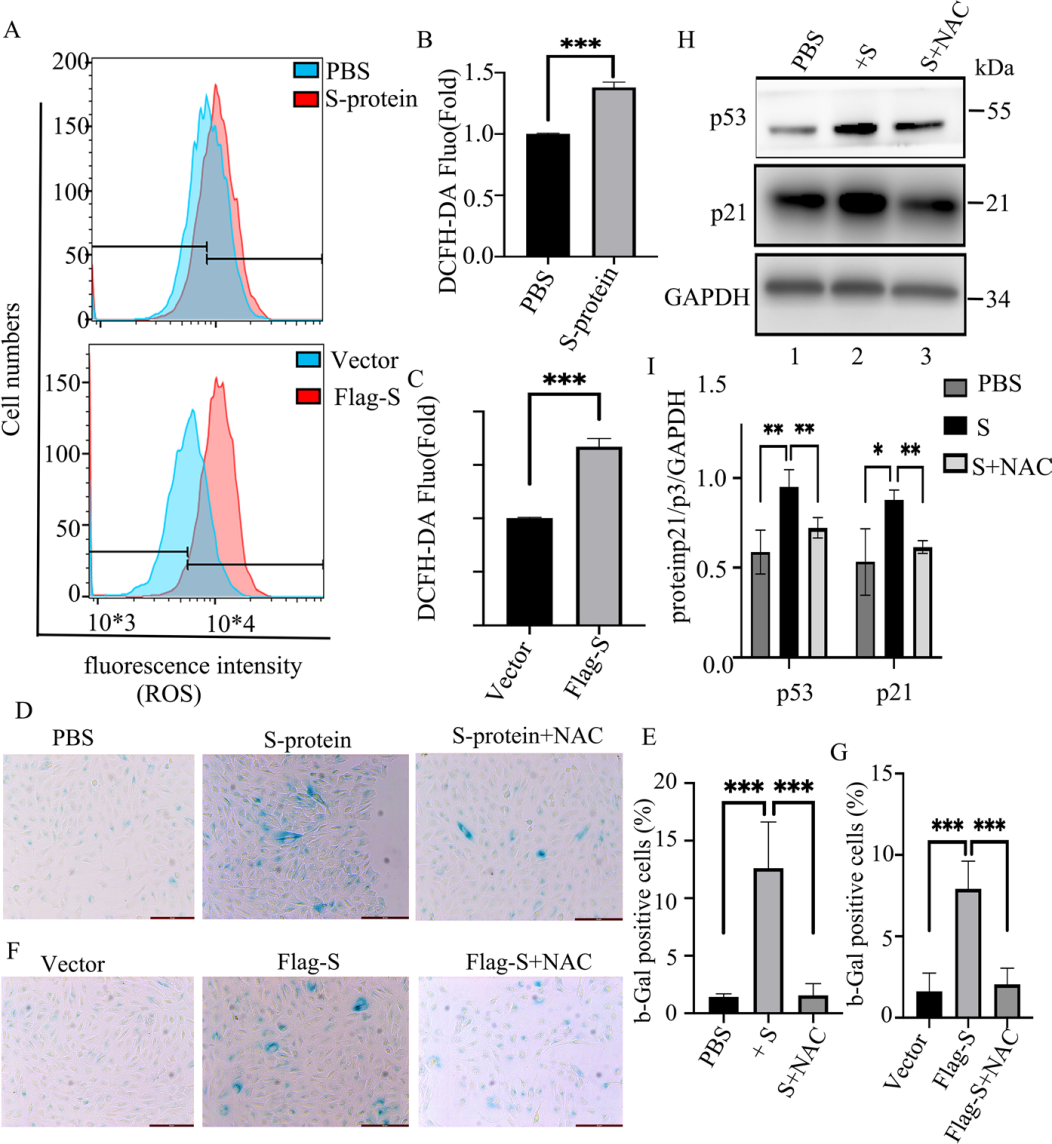
Removing ROS by NAC down-regulates S-protein-induced senescence of ARPE-19 cells. **A** Flow cytometry assay measuring DCFH-DA-degenerated fluorescence level in ARPE-19 cells that were treated with S-protein for 24 h (upper panel) or transfected with plasmid p3xFlag-S (lower panel). **B** and **C** Quantitation of DCFH-DA fluorescence in **A**. The data represent mean ± SD (n = 3), ***p < 0.001. **D** SA-β-Gal stain of ARPE cells that were treated with S-protein or S-protein + NAC for 48 h. **E** Quantitation of the percentage of SA-β-Gal positive cells in **D**. The data represent mean ± SD (n = 3), ***p < 0.001. **F** SA-β-Gal stain of ARPE-19 cells that were transfected with p3xflag-S followed by NAC treatment for 48 h. **G**, Quantitation of percentage of SA-β-Gal positive cells in **F**. The data represent means ± SD (n = 3), ***p < 0.001. **H**, immunoblot the expression of P53, P21 and GAPDH in ARPE-19 cells that were treated with media containing PBS (lane 1), S-protein (lane 2) and S-protein + NAC (lane 3)

Reactive oxygen species (ROS) is a predominant cause of cellular senescence under stress conditions.To determine whether ROS is involved in S-protein-induced cellular senescence, we measured ROS by adding DCFH-DA fluorescent dye to the 48 h-Flag-S-overexpressed or the 48 h-S-protein-treated ARPE-19 cells. The results showed that the ROS production increased in the cells at both treated conditions (Fig. 4A–C). Inhibition of ROS by NAC reduced the number of SA-β-Gal -positive cells (Fig. 4D–G) as well as the expression of p53 and p21 (Fig. 4H and I) in Flag-S-overexpressed or S-protein-treated ARPE-19 cells. These results indicated increased ROS is associated with S-protein-induced cellular senescence.

### S-protein induces ER stress in ARPE -19 cells

**Fig. 5:**
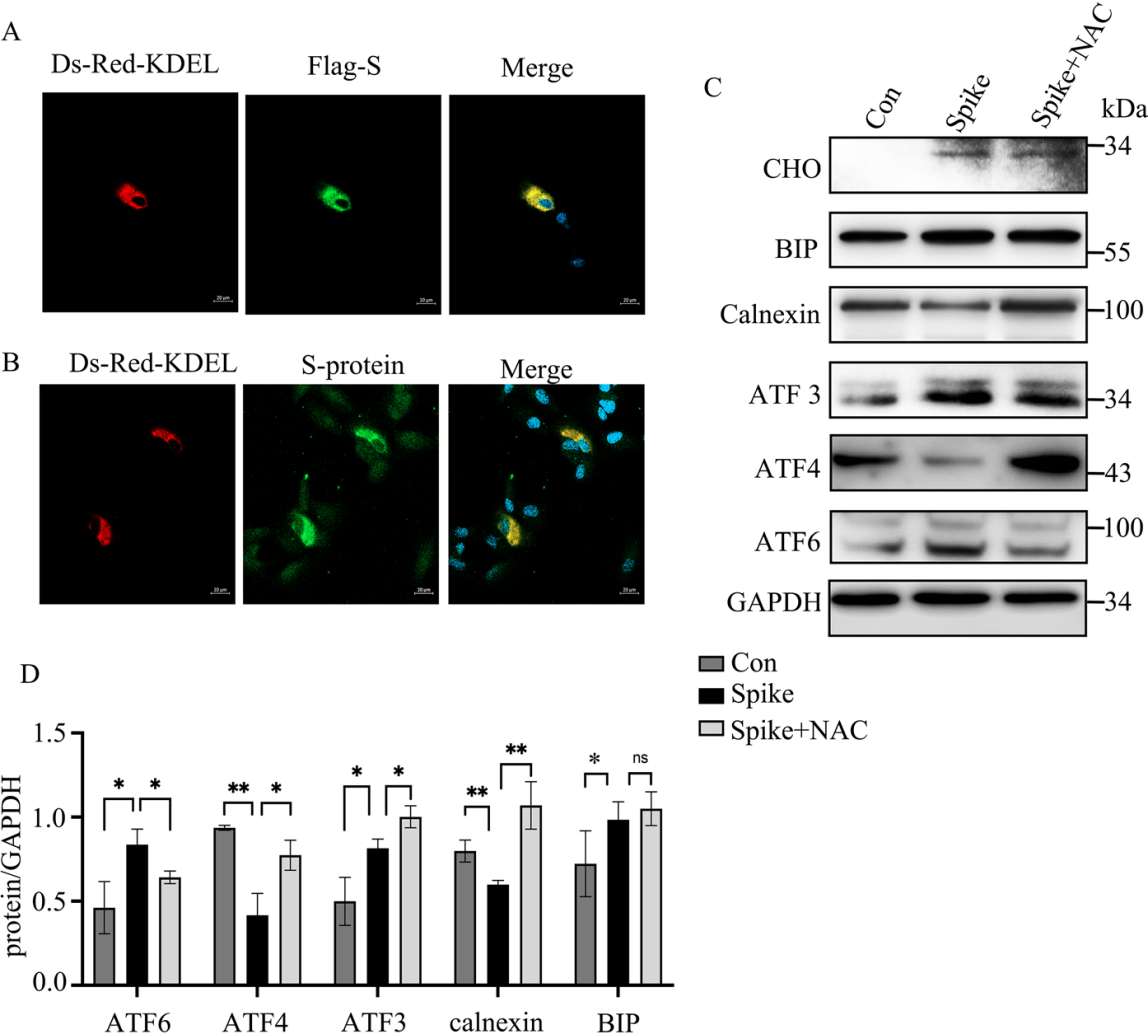
S-protein induces ER stress in ARPE-19 cells. **A** and **B** Immunofluorescence staining of S-protein in S-protein-treated (**B**) or Flag-S overexpressed ARPE-19 cells (**A**) that were transfected with pDs-Red-KDEL. **C** Immunoblot of the expression of CHOP, BIP, Calnexin, ATF3, ATF4, ATF6, and β-actin in ARPE-19 cells treated with S-protein or treated with S-protein S-protein + NAC for 24 h. **D** Densitometry quantitation of those proteins in **C**. The data represent mean ± SD (n = 3), *p < 0.05, **p < 0.01

The SASR-CoV-2 infection causes cellular ER stress, which is important for virus replication and host cell injury (Rashid et al. 2021; Santerre et al. 2021). We further studied the association of S-protein and ER stress in ARPE-19 cells. ARPE-19 cells were either transiently transfected with plasmid pKDEL-Ds-Red (to specifically label intracellular ER and Golgi) followed by incubating with S-protein for 30 min (Fig. 5A), or transiently co-transfected with pKDEL-Ds-Red and p3xflag-S for 48 h (Fig. 5B). The results of immunofluorescence assay indicated that both the overexpressed Flag-S-protein and administrated S-protein colocalized with ER (Fig. 5A and B). Administration of S-protein at 500 ng/ml S-protein for 24 h increased the expression of BIP, CHOP, ATF3, and cleaved ATF6 (Fig. 5C, lanes 1 and 2, and D), but slightly reduced the expression of calnexin and ATF4. To determine the association of ROS and ER stress, we added NAC to S-protein treated ARPE-19 cells. The results showed that NAC did not change the S-protein-induced expression of BIP, CHOP, and ATF3, but restored the expression of ATF4 and calnexin (Fig. 5C and D). These results suggested that S-protein colocalized with ER and induced ER stress, and this regulation was not associated with ROS induction in part.

### S-protein induces inflammatory factor expression via activating NF-κB in ARPE-19 cells

**Fig. 6:**
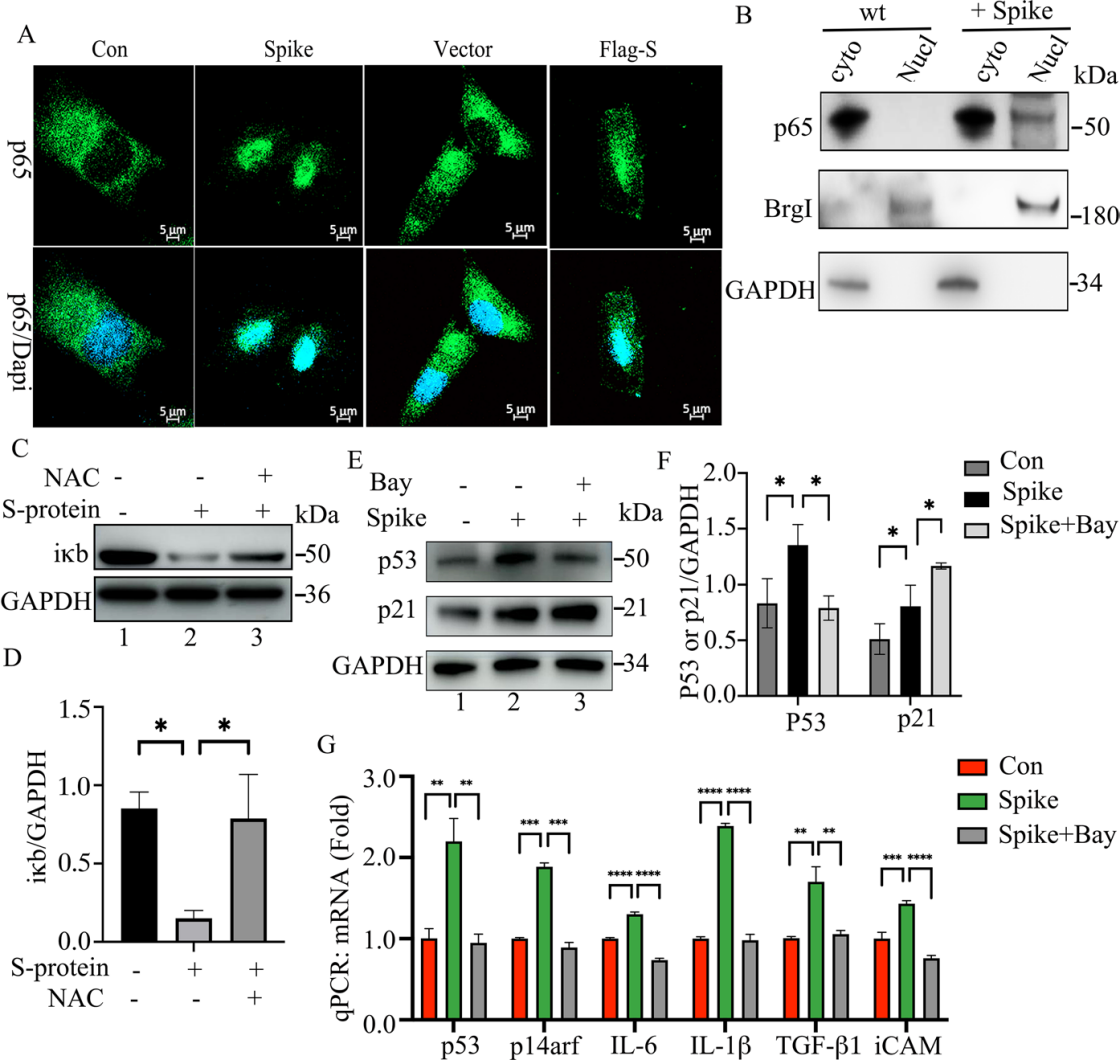
S-protein activates NF-κB pathways in ARPE-19 cells. **A** Immunofluorescence staining of p65 protein in S-protein-treated or Flag-S-overexpressed ARPE-19 cells. The nuclei were stained with DAPI. The scale bar represents 20 μm. **B** Cytosol-Nucleus fraction assay to determine the localization of p65 in ARPE-19 cells that were treated with PBS (Con) or S-protein for 48 h. BrgI and GAPDH were used as nuclear and cytosolic markers respectively. **C** Immunoblot of iκb and GAPDH in ARPE-19 cells that were treated with sham, S-protein and S-protein + NAC respectively. **D** Densitometry quantitation of iκb vs GAPDH in **C**. The data represent mean ± SD (n = 3), *p < 0.05. **E** Immunoblot of the expression of p53, p21 and GAPDH in ARPE-19 cells that were treated with sham, S-protein and S-protein + Bay-11–7082 (NF-κB inhibitor) for 24 h. **F** Densitometry quantitation of P53 and p21 in **E**. The P53 and P21 protein level was normalized to GAPDH. The data represent mean ± SD (n = 3), *p < 0.05. **G** qPCR measuring the expression of p53, p14arf, IL-1β, IL-6, TGF-β1 and iCAM in ARPE-19 cells treated in **E**. The data represent mean ± SD (n = 3), **p < 0.01, ***p < 0.001

Activation of the NF-κB pathway is closely associated with SASR-CoV-2-induced acute inflammation or the expression of senescence-associated inflammatory factors (Goel et al. 2021; Zaffagni et al. 2022). We further studied the activity of NF-κB in S-protein-treated ARPE-19 cells by using immunofluorescence and immunoblotting assays. The results showed that ectopic expression of Flag-S or administration of S-protein for 48 h increased P65 nuclear translocation, a symbol of NF-κB activation (Fig. 6A and B). Administration of S-protein reduced iκB protein expression, and this reduction of iκB protein was inhibited by ROS scavenger NAC (Fig. 6C and D). These results suggested that S-protein activated the NF-κB pathway by inducing ROS. Furthermore, we found that inhibition of NF-κB by inhibitor Bay-11–7082 at 10 μM for 30 min reduced S-protein-induced mRNA expression of p53, p14arf, IL-1β, IL-6, TGF-β1, and iCAM 1 as well as P53 protein expression, but not p21 expression (Fig. 6E, F, and G). These results suggested that S-protein stimulated senescence-associated cytokines by inducing ROS-activated NF-κB pathway, which is consistent with other reports (Chen et al. 2021).

### S-protein upregulates the expression of senescence-associated cytokines in zebrafish Retina

**Fig. 7:**
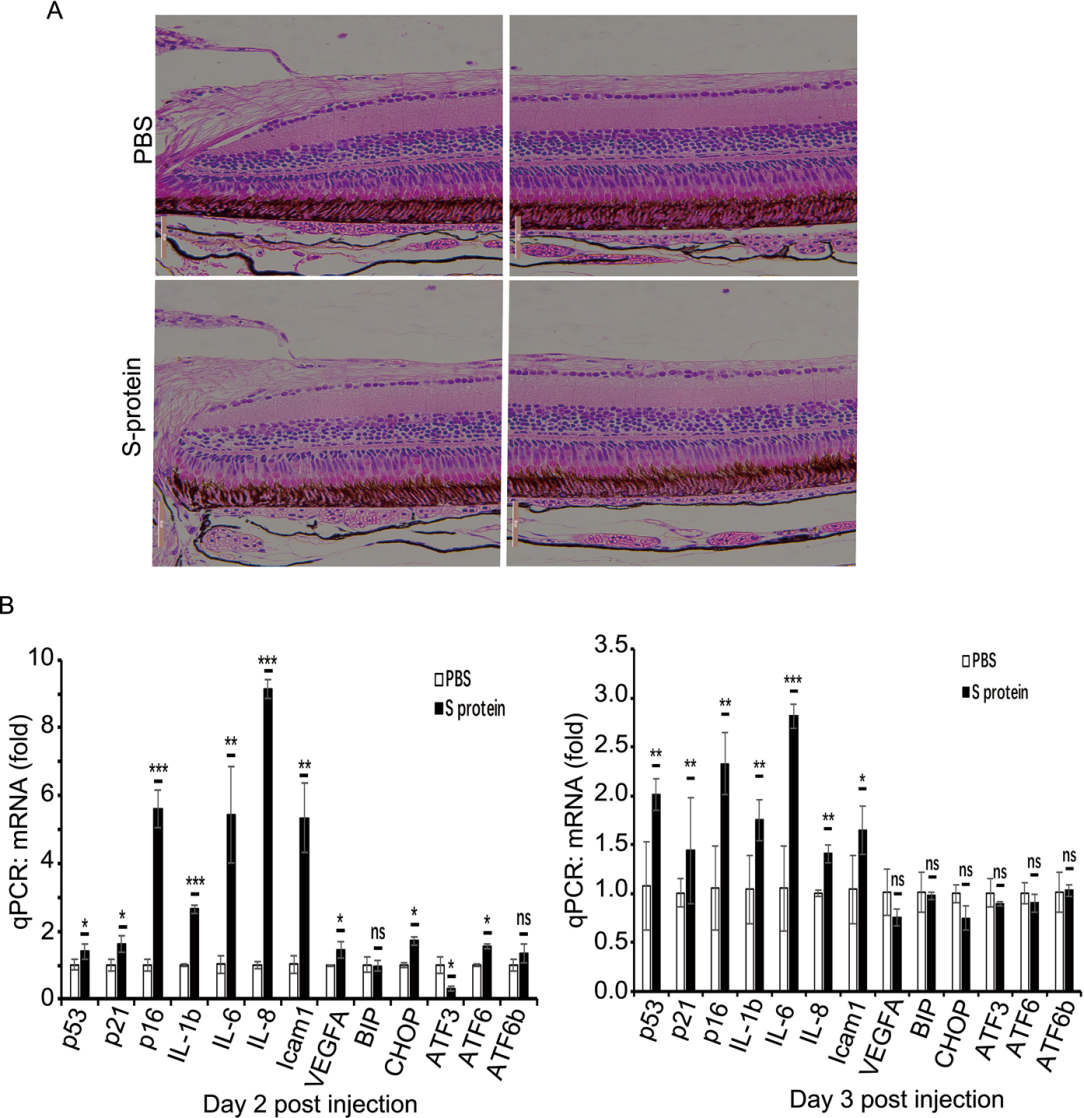
S-protein induces the expression of senescence-associated factors in the zebrafish retina. **A** H&E analysis of day 2 zebrafish retina post injection of PBS or 6.3 ng S-protein. **B** Quantitative RT-PCR measuring the expression of P53, P21, P16, IL-1β, IL-6, IL-8, ICAM1, VEGF-A, BIP, CHOP, ATF3, ATF6 in day 2 and day 3 recovered zebrafish retina post injection of S-proteins. The data showed mean ± SD, n = 6. The unpaired student t-test was used for statistical analysis. *p < 0.05, **p < 0.01

Next, we studied the cytotoxicity of S-protein to the retina in a zebrafish model. Purified S-protein (6.3 ng/100 nl PBS) was intravitreally injected into the right eye of 7 mpf zebrafish followed by recovery for 2 and 3 days. An equal amount of PBS was injected into the left eye as a control. The retina was cryosected followed by H&E staining. The results showed that S-protein did not change the structure of the retina compared to PBS (Fig. 7A). TUNEL assay showed no DNA damage in both S-protein- and PBS- treated retina (data not shown). However, the qPCR results showed that S-protein upregulated senescence-associated genes’ mRNA expression such as P53, P21, P16, IL-1β, IL-6, IL-8 and ICAM-1 in both day 2 and 3 recovery retina compared to PBS. No obvious change in the expression of UPR-associated protein such as BIP, ATG6, CHOP, and ATF3 (Fig. 7B and C). These results demonstrated that administration of S-protein trigger the senescent phenotype in zebrafish retinal tissues.

## Discussion

SARS-CoV-2 infection attacks multiple organs, leading to acute inflammation(Guan et al. 2020) as well as the chronic complications such as chronic fibrosis in kidney and lung(Bui et al. 2021; Jansen et al. 2022). Aging increases SARS-CoV-2 infection incidence and inflammatory symptoms. On the other hand, infection of SARS-CoV-2 and its spike protein can trigger cellular senescence, a cellular process associated with ageing and age-associated diseases such as tumor, neurodegenerative diseases, diabetics and AMD (Collado et al. 2007; Meyer et al. 2021; Tripathi et al. 2021). Here we show that the S-protein of SARS-CoV-2 can induce ARPE-19 cell senescence in vitro by upregulating cellular ROS, ER stress, and NF-κB pathways (Figs. 3, 4, 5 and 6). Intravitreal administration of S-protein upregulates the expression of senescence-associated inflammatory factors in the zebrafish retina (Fig. 7). These results reveal a potential association of SARS-CoV-2 infection and retinopathy.

Accumulating evidence demonstrate that increased number of senescent RPE cells are associated with AMD by producing senescence-associated cytokines, injuring the retinal barrier, and losing its endocytosis ability or vision cycle (Hjelmeland et al. 1999; Ambati et al. 2003). The virus infection is coorelated with retinopathy, including development of AMD. For example, infection by latent CMV has been shown to promote neovascularization in VEGFA-overexpressed mice (Xu et al. 2020). Infection of HerPs/-virus -6A may be another virulent contributor to the development of AMD by downregulating complement receptor CD46 in RPE and choroid endothelial cells (Fierz 2017). Our data suggest that the S-protein of SARS-CoV-2 may be a virulent factor in triggering retinopathy. We find that both administration of purified S-protein and ectopic expression of Flag-S-protein induce ARPE-19 cell senescence (Figs. 1 and 2). This is consistent with previous report demonstrating that the Spike protein can cause senescence in lung tumor cells in vitro (Tripathi et al. 2021). The senescence induction by S-protein is associated with upregulation of ROS production (Fig. 3). Removing ROS by NAC reduces S-protein-induced cellular senescence and secretion of cytokines IL-1 and IL-6 (Fig. 3). SARS-Cov-2 has been shown to increase ROS by decreasing the glutathione (GSH) and increasing GSSG in infected cells. It accomplishes this by increasing GSH efflux and/or decreasing Cysteine uptake (Bartolini et al. 2021). Our data suggest that the spike protein is one of the pathogenic factors of SARS-CoV that increases ROS.

Activation ER stress is also involved in SARS-Cov-2-induced pathological change (Bartolini et al. 2022). SARS-Cov-2 virus activates the PERK/IRE pathway via S-protein interacting with ER (Versteeg et al. 2007). We find both ectopic expression and administration of S-protein colocalized with the ER and activated ER stress by possibly activating ATF6 (Fig. 5). S protein contains ER retention peptide, and its ER localization is regulated by viral membrane proteins E and M (Boson et al. 2021). However, the molecular mechanism underlying the traffic of administrated S-protein to ER remain unclear. Whether its receptor proteins such as laminin(Bamberger et al. 2021), ACE2, BIP are involved in the traffic of extracellular S-protein to ER remain unclear.

In addition, we studied the role of S-protein in retina by artificially administering S-protein into zebrafish vitreous humor, and found that S-protein can induce the mRNA expression of senescence-associated genes without impairing retina structure (Fig. 7). We did not observed DNA damage in S-protein treated zebrafish retina (data not shown). This suggest the administrated S-protein can trigger the senescent phenotype, but which cell type in retina undergo senescence still remain unclear. In this zebrafish model, we only administrated S-protein once, and observation time is short. Prolonged observation of the regulatory effects of S-protein on retinopathy is necessary, which is still under investigation.

## Conclusions

SARS-COV-2 spike protein can induce ARPE-19 cells to undergo senescence by increasing cellular ROS or ER stress in vitro. The SARS-COV-2 spike protein may be associated with development of chronic retinal degeneration.

## Supplementary Information

Below is the link to the electronic supplementary material.

Supplementary file1 (DOCX 261 kb)

## Data Availability

The data used to support the findings of this study are included within the article.

## References

[CR1] Ambati J, Anand A, Fernandez S, Sakurai E, Lynn BC, Kuziel WA, Rollins BJ, Ambati BK (2003). An animal model of age-related macular degeneration in senescent Ccl-2- or Ccr-2-deficient mice. Nat Med.

[CR2] Araujo-Silva CA, Marcos AAA, Marinho PM, Branco AMC, Roque A, Romano AC, Matuoka ML, Farah M, Burnier M, Moraes NF, Tierno P, Schor P, Sakamoto V, Nascimento H, de Sousa W, Belfort R (2021). Presumed SARS-CoV-2 viral particles in the human retina of patients with COVID-19. JAMA Ophthalmol.

[CR3] Bamberger C, Pankow S, Martínez-Bartolomé S, Diedrich J, Park R, Yates J (2021). The host interactome of spike expands the tropism of SARS-CoV-2. bioRxiv.

[CR4] Bartolini D, Stabile AM, Bastianelli S, Giustarini D, Pierucci S, Busti C, Vacca C, Gidari A, Francisci D, Castronari R, Mencacci A, Di Cristina M, Focaia R, Sabbatini S, Rende M, Gioiello A, Cruciani G, Rossi R, Galli F (2021). SARS-CoV2 infection impairs the metabolism and redox function of cellular glutathione. Redox Biol.

[CR5] Bartolini D, Stabile AM, Vacca C, Pistilli A, Rende M, Gioiello A, Cruciani G, Galli F (2022). Endoplasmic reticulum stress and NF-kB activation in SARS-CoV-2 infected cells and their response to antiviral therapy. IUBMB Life.

[CR6] Bonn D (2004). SARS virus in tears?. Lancet Infect Dis.

[CR7] Boson B, Legros V, Zhou B, Siret E, Mathieu C, Cosset FL, Lavillette D, Denolly S (2021). The SARS-CoV-2 envelope and membrane proteins modulate maturation and retention of the spike protein, allowing assembly of virus-like particles. J Biol Chem.

[CR8] Bourne RR, Stevens GA, White RA, Smith JL, Flaxman SR, Price H, Jonas JB, Keeffe J, Leasher J, Naidoo K, Pesudovs K, Resnikoff S, Taylor HR (2013). Causes of vision loss worldwide, 1990–2010: a systematic analysis. Lancet Glob Health.

[CR9] Bui LT, Winters NI, Chung MI, Joseph C, Gutierrez AJ, Habermann AC, Adams TS, Schupp JC, Poli S, Peter LM, Taylor CJ, Blackburn JB, Richmond BW, Nicholson AG, Rassl D, Wallace WA, Rosas IO, Jenkins RG, Kaminski N, Kropski JA, Banovich NE, N. Human Cell Atlas Lung Biological (2021). Chronic lung diseases are associated with gene expression programs favoring SARS-CoV-2 entry and severity. Nat Commun.

[CR10] Camell CD, Yousefzadeh MJ, Zhu Y, Prata L, Huggins MA, Pierson M, Zhang L, O'Kelly RD, Pirtskhalava T, Xun P, Ejima K, Xue A, Tripathi U, Espindola-Netto JM, Giorgadze N, Atkinson EJ, Inman CL, Johnson KO, Cholensky SH, Carlson TW, LeBrasseur NK, Khosla S, O'Sullivan MG, Allison DB, Jameson SC, Meves A, Li M, Prakash YS, Chiarella SE, Hamilton SE, Tchkonia T, Niedernhofer LJ, Kirkland JL, Robbins PD (2021). Senolytics reduce coronavirus-related mortality in old mice. Science.

[CR11] Chen DD, Peng X, Wang Y, Jiang M, Xue M, Shang G, Liu X, Jia X, Liu B, Lu Y, Mu H, Zhang F, Hu Y (2021). HSP90 acts as a senomorphic target in senescent retinal pigmental epithelial cells. Aging (albany NY).

[CR12] Cheng K-C, Hsu Y-T, Liu W, Huang H-L, Chen L-Y, He C-X, Sheu S-J, Chen K-J, Lee P-Y, Lin Y-H, Chiu C-C (2021). The role of oxidative stress and autophagy in blue-light-induced damage to the retinal pigment epithelium in zebrafish in vitro and in vivo. Int J Mol Sci.

[CR13] Collado M, Blasco MA, Serrano M (2007). Cellular senescence in cancer and aging. Cell.

[CR14] Dimitrov DS (2003). The secret life of ACE2 as a receptor for the SARS virus. Cell.

[CR15] Duarte C, Akkaoui J, Ho A, Garcia C, Yamada C, Movila A (2021). Age-dependent effects of the recombinant spike protein/SARS-CoV-2 on the M-CSF- and IL-34-differentiated macrophages in vitro. Biochem Biophys Res Commun.

[CR16] Faragher RGA (2021). Simple detection methods for senescent cells: opportunities and challenges. Front Aging.

[CR17] Fierz W (2017). Age-related macular degeneration: a connection between human herpes virus-6A-induced CD46 downregulation and complement activation?. Front Immunol.

[CR18] Goel S, Saheb Sharif-Askari F, Saheb Sharif Askari N, Madkhana B, Alwaa AM, Mahboub B, Zakeri AM, Ratemi E, Hamoudi R, Hamid Q, Halwani R (2021). SARS-CoV-2 switches 'on' MAPK and NFkappaB signaling via the reduction of nuclear DUSP1 and DUSP5 expression. Front Pharmacol.

[CR19] Guan WJ, Ni ZY, Hu Y, Liang WH, Ou CQ, He JX, Liu L, Shan H, Lei CL, Hui DSC, Du B, Li LJ, Zeng G, Yuen KY, Chen RC, Tang CL, Wang T, Chen PY, Xiang J, Li SY, Wang JL, Liang ZJ, Peng YX, Wei L, Liu Y, Hu YH, Peng P, Wang JM, Liu JY, Chen Z, Li G, Zheng ZJ, Qiu SQ, Luo J, Ye CJ, Zhu SY, Zhong NS, C. China Medical Treatment Expert Group for (2020). Clinical characteristics of coronavirus disease 2019 in China. N Engl J Med.

[CR20] Hamming I, Timens W, Bulthuis ML, Lely AT, Navis G, van Goor H (2004). Tissue distribution of ACE2 protein, the functional receptor for SARS coronavirus. A first step in understanding SARS pathogenesis. J Pathol.

[CR21] Heurich A, Hofmann-Winkler H, Gierer S, Liepold T, Jahn O, Pohlmann S (2014). TMPRSS2 and ADAM17 cleave ACE2 differentially and only proteolysis by TMPRSS2 augments entry driven by the severe acute respiratory syndrome coronavirus spike protein. J Virol.

[CR22] Hjelmeland LM, Cristofolo VJ, Funk W, Rakoczy E, Katz ML (1999). Senescence of the retinal pigment epithelium. Mol vis.

[CR23] Hoffmann M, Kleine-Weber H, Schroeder S, Kruger N, Herrler T, Erichsen S, Schiergens TS, Herrler G, Wu NH, Nitsche A, Muller MA, Drosten C, Pohlmann S (2020). SARS-CoV-2 cell entry depends on ACE2 and TMPRSS2 and is blocked by a clinically proven protease inhibitor. Cell.

[CR24] Jansen J, Reimer KC, Nagai JS, Varghese FS, Overheul GJ, de Beer M, Roverts R, Daviran D, Fermin LAS, Willemsen B, Beukenboom M, Djudjaj S, von Stillfried S, van Eijk LE, Mastik M, Bulthuis M, Dunnen WD, van Goor H, Hillebrands JL, Triana SH, Alexandrov T, Timm MC, van den Berge BT, van den Broek M, Nlandu Q, Heijnert J, Bindels EMJ, Hoogenboezem RM, Mooren F, Kuppe C, Miesen P, Grünberg K, Ijzermans T, Steenbergen EJ, Czogalla J, Schreuder MF, Sommerdijk N, Akiva A, Boor P, Puelles VG, Floege J, Huber TB, van Rij RP, Costa IG, Schneider RK, Smeets B, Kramann R (2022). SARS-CoV-2 infects the human kidney and drives fibrosis in kidney organoids. Cell Stem Cell.

[CR25] Jonas JB, Cheung CMG, Panda-Jonas S (2017). Updates on the epidemiology of age-related macular degeneration. Asia Pac J Ophthalmol (phila).

[CR26] Kodaka M, Yang Z, Nakagawa K, Maruyama J, Xu X, Sarkar A, Ichimura A, Nasu Y, Ozawa T, Iwasa H, Ishigami-Yuasa M, Ito S, Kagechika H, Hata Y (2015). A new cell-based assay to evaluate myogenesis in mouse myoblast C2C12 cells. Exp Cell Res.

[CR27] Lani-Louzada R, Ramos C, Cordeiro RM, Sadun AA (2020). Retinal changes in COVID-19 hospitalized cases. PLoS ONE.

[CR28] Li W, Moore MJ, Vasilieva N, Sui J, Wong SK, Berne MA, Somasundaran M, Sullivan JL, Luzuriaga K, Greenough TC, Choe H, Farzan M (2003). Angiotensin-converting enzyme 2 is a functional receptor for the SARS coronavirus. Nature.

[CR29] Maremanda KP, Sundar IK, Li D, Rahman I (2020). Age-dependent assessment of genes involved in cellular senescence, telomere and mitochondrial pathways in human lung tissue of smokers, COPD and IPF: associations with SARS-CoV-2 COVID-19 ACE2-TMPRSS2-Furin-DPP4 axis. Front Pharmacol.

[CR30] Marinho PM, Marcos AAA, Romano AC, Nascimento H, Belfort R (2020). Retinal findings in patients with COVID-19. Lancet.

[CR31] Martin G, Wolf J, Lapp T, Agostini HT, Schlunck G, Auw-Hadrich C, Lange CAK (2021). Viral S protein histochemistry reveals few potential SARS-CoV-2 entry sites in human ocular tissues. Sci Rep.

[CR32] Meyer K, Patra T, Vijayamahantesh, Ray R (2021). SARS-CoV-2 spike protein induces paracrine senescence and leukocyte adhesion in endothelial cells. J Virol.

[CR33] Rashid F, Dzakah EE, Wang H, Tang S (2021). The ORF8 protein of SARS-CoV-2 induced endoplasmic reticulum stress and mediated immune evasion by antagonizing production of interferon beta. Virus Res.

[CR34] Reinhold A, Tzankov A, Matter MS, Mihic-Probst D, Scholl HPN, Meyer P (2021). Ocular pathology and occasionally detectable intraocular severe acute respiratory syndrome coronavirus-2 RNA in five fatal coronavirus disease-19 cases. Ophthalmic Res.

[CR35] Santerre M, Arjona SP, Allen CN, Shcherbik N, Sawaya BE (2021). Why do SARS-CoV-2 NSPs rush to the ER?. J Neurol.

[CR36] Schuler BA, Habermann AC, Plosa EJ, Taylor CJ, Jetter C, Negretti NM, Kapp ME, Benjamin JT, Gulleman P, Nichols DS, Braunstein LZ, Hackett A, Koval M, Guttentag SH, Blackwell TS, Webber SA, Banovich NE, Vanderbilt C-CC, Kropski JA, Sucre JM, N. Human Cell Atlas Biological (2021). Age-determined expression of priming protease TMPRSS2 and localization of SARS-CoV-2 in lung epithelium. J Clin Invest.

[CR37] Song W, Gui M, Wang X, Xiang Y (2018). Cryo-EM structure of the SARS coronavirus spike glycoprotein in complex with its host cell receptor ACE2. PLoS Pathog.

[CR38] Tripathi U, Nchioua R, Prata L, Zhu Y, Gerdes EOW, Giorgadze N, Pirtskhalava T, Parker E, Xue A, Espindola-Netto JM, Stenger S, Robbins PD, Niedernhofer LJ, Dickinson SL, Allison DB, Kirchhoff F, Sparrer KMJ, Tchkonia T, Kirkland JL (2021). SARS-CoV-2 causes senescence in human cells and exacerbates the senescence-associated secretory phenotype through TLR-3. Aging (albany NY).

[CR39] Upadhyay M, Milliner C, Bell BA, Bonilha VL (2020). Oxidative stress in the retina and retinal pigment epithelium (RPE): role of aging, and DJ-1. Redox Biol.

[CR40] Urbanelli L, Buratta S, Sagini K, Tancini B, Emiliani C (2016). Extracellular vesicles as new players in cellular senescence. Int J Mol Sci.

[CR41] Versteeg GA, van de Nes PS, Bredenbeek PJ, Spaan WJ (2007). The coronavirus spike protein induces endoplasmic reticulum stress and upregulation of intracellular chemokine mRNA concentrations. J Virol.

[CR42] Virgo J, Mohamed M (2020). Paracentral acute middle maculopathy and acute macular neuroretinopathy following SARS-CoV-2 infection. Eye (lond).

[CR43] Wan KH, Huang SS, Lam DSC (2021). Conjunctival findings in patients with coronavirus disease 2019. JAMA Ophthalmol.

[CR44] Xu J, Liu X, Zhang X, Marshall B, Dong Z, Liu Y, Espinosa-Heidmann DG, Zhang M (2020). Ocular cytomegalovirus latency exacerbates the development of choroidal neovascularization. J Pathol.

[CR45] Yan R, Zhang Y, Li Y, Xia L, Guo Y, Zhou Q (2020). Structural basis for the recognition of the SARS-CoV-2 by full-length human ACE2. Science.

[CR46] Zaffagni M, Harris JM, Patop IL, Pamudurti NR, Nguyen S, Kadener S (2022). SARS-CoV-2 Nsp14 mediates the effects of viral infection on the host cell transcriptome. Elife.

[CR47] Zhou P, Yang XL, Wang XG, Hu B, Zhang L, Zhang W, Si HR, Zhu Y, Li B, Huang CL, Chen HD, Chen J, Luo Y, Guo H, Jiang RD, Liu MQ, Chen Y, Shen XR, Wang X, Zheng XS, Zhao K, Chen QJ, Deng F, Liu LL, Yan B, Zhan FX, Wang YY, Xiao GF, Shi ZL (2020). A pneumonia outbreak associated with a new coronavirus of probable bat origin. Nature.

