# Supplementary material for: SARS-COV-2 spike protein promotes RPE cell senescence via the ROS/P53/P21 pathway
Source: Biogerontology. 2023 Feb 4;24(5):813–27. doi: 10.1007/s10522-023-10019-0 (PMC9898700; doi:10.1007/s10522-023-10019-0)
Supplement: Supplementary file 1 — Supplementary file1 (DOCX 261 kb) [file 10522_2023_10019_MOESM1_ESM.docx]

SARS-COV-2 Spike Protein Promotes RPE Cell Senescence via the ROS/P53/P21 Pathway

Yuhang Zhang^1a^, Xuyan Peng^1a^, Mengjiao Xue^1a^, Jingjing Liu^1^, Guohui Shang^4^, Mingjun Jiang^1^, Dandan Chen^1^, Baixue Liu^1^, Yuxuan Wang^1^, Xiaolin Jia^1^, Jianqing Xu^5^, Fengyan Zhang^1*^, Yanzhong Hu^1,2,3*^

^1^The Laboratory of Ophthalmology and Vision Science, Department of Ophthalmology, The First Affiliated Hospital of Zhengzhou University, Zheng Zhou, China

^2^Joint National Laboratory for Antibody Drug Engineering, The First affiliated hospital of Henan University. Kaifeng, China

^3^Kaifeng Key Lab for Cataract and Myopia, Institute of Eye Disease, Kaifeng Central Hospital, Kaifeng, China

^4^Department of Medical Genetics and Cell Biology, School of Basic Medical Sciences, Zhengzhou University, Zheng Zhou, China

^5^Chongqing Institutes for Life Science Innovation; Clinical Center for Bio-therapy, Zhongshan Hospital & Institutes of Biomedical Sciences, Fudan University, Shanghai 200032, P.R. China.

Running title: SARS-Cov-2 S-protein induces RPE cell senescence.

^a^These authors contributed equally to this work

* Corresponding author:

Yanzhong Hu, professor, (hyz@henu.edu.cn) Department of Cell Biology and Genetics, School of Medicine, Henan University, Jin-Ming Road, Kaifeng, China, 475014; Tel. 86-18503781944.

Fengyan Zhang，Ph.D./ M.D. (Zhangfengyanx@aliyun.com). The Division of Ophthalmology and Vision Science, Department of Ophthalmology, The First Affiliated Hospital of Zhengzhou University, Zhengzhou University, No.1 Long-Hu-Zhong Huan Road, Zhengzhou, China

**
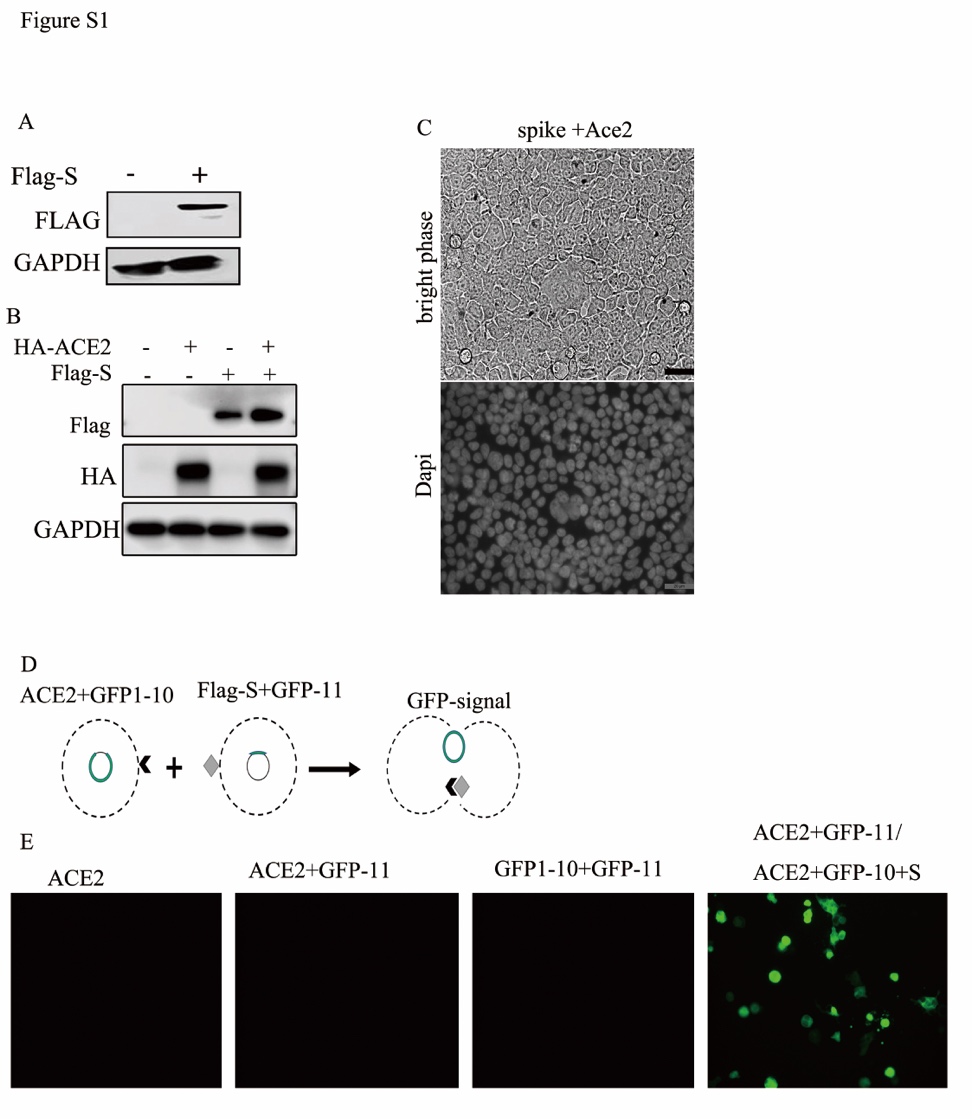
**

**Figure S1**.

Figure S1, spike protein induces HEK293 cells fusion. A, immunoblotting the expression of Flag-S and GAPDH in HEK293 cells that were transfected with empty vector (lane 1) or p3xflag-S (lane 2). B, immunoblotting the overexpressed Flag-S or HA-ACE2 in HEK293 cells. C, Fusion of HEK293/Flag-S and HEK293/HA-ACE2 cells for 48hours. upper penal is the phase-contrast photograph, the low panel is Dapi staining for cell nuclei. D, the schematic map of BiFC assay to measure cell fusion. pGFP1-10 and pGFPp1-11 interacts with each other to generate green GFP signal. E, GFP-indicated cell fusion assay (BiFC), fluorescent microscope used to detect the GFP fluorescence in HEK293/ HA-ACE2 only, HEK293/HA-ACE2/GFP1-10, HEK293/Flag-S/GFP-11 or co-cultured HEK293/ACE2+GFP1-10 with HEK293/Flag-S+GFP-11.

**
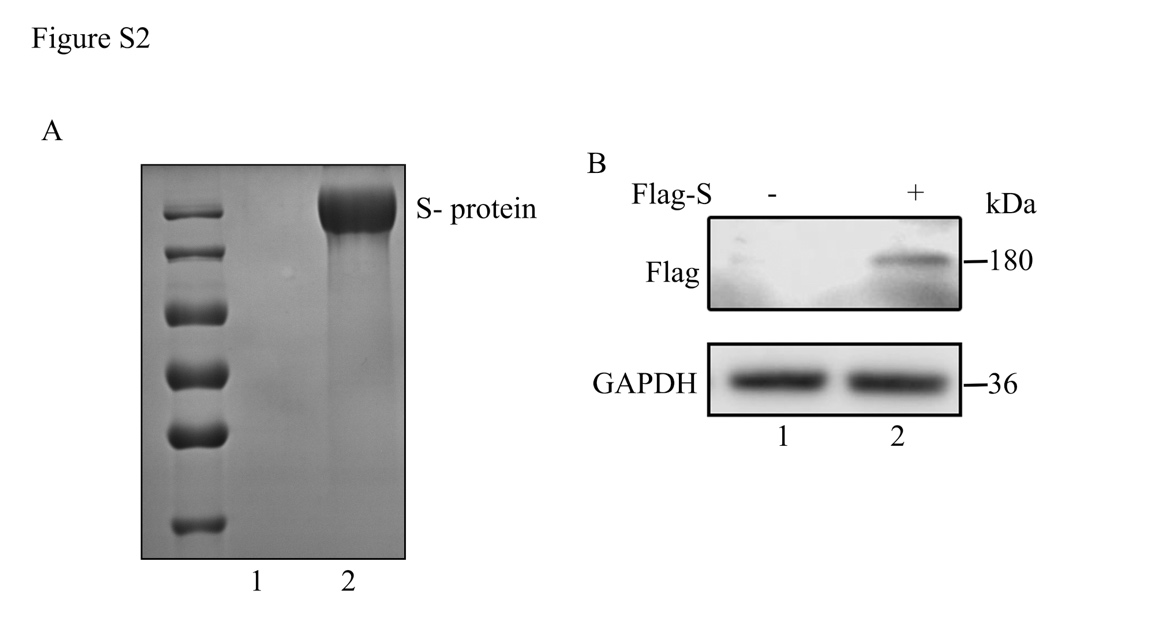
**

**Figure S2.**

The Spike protein used to treat ARPE -19 cells. A, Coomassie blue staining the spike protein in full length ( lane 2). B, the plasmid of p3xflag-Spike-cmv-7.1 was transfected into HEK293 cells and the expression of the tagged proteins was detected (lane 2). GAPDH protein was used as the protein loading control.
